# Spatial immune ecology of immunotherapy resistance in gastric and gastroesophageal junction adenocarcinoma

**DOI:** 10.3389/fonc.2026.1895422

**Published:** 2026-07-21

**Authors:** Xinri Zhang, Xingyu Chen, Yan Leng

**Affiliations:** 1 College of Traditional Chinese Medicine, Changchun University of Traditional Chinese Medicine, Changchun, Jilin, China; 2 Department of Liver, Spleen and Gastroenterology, Affiliated Hospital of Changchun University of Traditional Chinese Medicine, Changchun, Jilin, China

**Keywords:** gastric adenocarcinoma, gastroesophageal junction adenocarcinoma, immune checkpoint inhibitors, immunotherapy resistance, spatial biomarkers, spatial immune ecology, tertiary lymphoid structures, tumor microenvironment

## Abstract

Immune checkpoint inhibitors now occupy a central place in the management of advanced gastric and gastroesophageal junction adenocarcinoma, yet durable benefit remains uneven across patients, lesions, and metastatic sites. Conventional biomarkers, including programmed death-ligand 1 scoring, microsatellite instability or mismatch-repair deficiency, HER2, claudin 18.2, Epstein-Barr virus status, and tumor mutational burden, define important clinical and biological contexts but do not fully explain whether antitumor immunity can reach, recognize, and control malignant tissue. This narrative Review uses a spatial immune-ecology lens to interpret immunotherapy resistance through three linked tissue-level axes: immune accessibility, immune competence, and immune suppression. Within this framework, gastric and gastroesophageal junction adenocarcinoma can be organized into immune-desert, immune-excluded, inflamed-dysfunctional, tertiary lymphoid structure-rich immune-active, and metastatic-niche ecosystems, with particular emphasis on peritoneal and liver metastases. We distinguish gastric or gastric/gastroesophageal junction immune-checkpoint-inhibitor outcome-linked evidence from gastric spatial-biology or prognostic evidence, pan-cancer mechanisms, and more exploratory concepts. For now, spatial features such as tumor-nest CD8+ infiltration, tertiary lymphoid structure maturity, LAMP3+ dendritic-cell proximity, cancer-associated fibroblast-myeloid barriers, and lesion-level biomarker discordance are best regarded as complements to guideline-supported biomarkers and as pharmacodynamic endpoints, rather than independent treatment-selection tools, until they are tested prospectively in gastric and gastroesophageal junction immune-checkpoint-inhibitor outcome-linked cohorts.

## Introduction

Gastric cancer remains a major source of cancer morbidity and mortality worldwide, and advanced gastric or gastroesophageal junction adenocarcinoma still achieves durable disease control in only a subset of patients despite recent therapeutic advances (1). Molecular profiling has made clear that gastric adenocarcinoma is not a single biological entity. The Cancer Genome Atlas and Asian Cancer Research Group classifications delineate EBV-positive, MSI, genomically stable, chromosomally unstable, and other clinically relevant subgroups, each with distinct patterns of antigenicity, stromal composition, epithelial lineage state, metastatic behavior, and immune contexture (2, 3).

Immune checkpoint inhibitors have reshaped the treatment landscape in advanced disease. Phase III trials support PD-1 blockade combined with chemotherapy, and HER2-positive disease has become an instructive setting in which checkpoint blockade is integrated with targeted therapy (4–9). The clinical responses, however, are far from uniform. Some tumors never respond, some adapt during treatment, and others progress after an initial period of disease control. This spectrum reflects primary, adaptive, and acquired resistance, driven by tumor-intrinsic programs as well as microenvironmental constraints (10, 11).

Current biomarkers are indispensable but incomplete. PD-L1 combined positive score (CPS) or Tumor Area Positivity (TAP) score, microsatellite instability or mismatch-repair deficiency (MSI/dMMR), HER2, and CLDN18.2 are clinically established or increasingly implemented in defined treatment contexts, whereas Epstein-Barr virus (EBV) status and tumor mutational burden (TMB) add biological and, in some settings, supportive interpretive value. None of these markers, by itself, establishes whether cytotoxic lymphocytes can enter malignant epithelial nests, whether antigen-presenting cells are positioned to restimulate T cells, or whether stromal, vascular, metabolic, and myeloid barriers prevent immune engagement (10–12). The clinically relevant question is therefore not simply whether immune-relevant molecules or cells are present, but where they are located and whether they can interact productively.

The same logic is embedded in broader concepts of immune contexture, immunoediting, and immune hot, altered, and cold tumor states (13–17). Single-cell RNA sequencing has expanded the gastric cancer field by resolving malignant epithelial states, exhausted and progenitor-like T cells, Tregs, B-cell and plasma-cell programs, dendritic-cell subsets, tumor-associated macrophages, neutrophils, CAFs, endothelial cells, and perivascular cells (18–22). Spatial transcriptomic, spatial proteomic, and multiplex imaging studies now supply the anatomical coordinates needed to place these states within tumor nests, stromal margins, lymphoid aggregates, invasive fronts, and metastatic niches (23–30).

In this Review, we interpret immunotherapy resistance in gastric and gastroesophageal junction adenocarcinoma as a spatially organized tissue-ecological phenotype rather than the simple consequence of isolated molecular biomarkers or individual immune-cell subsets. We use spatial immune ecology to mean the tissue-level organization and interaction of malignant epithelial, immune, stromal, vascular, and metastatic-niche compartments that determine whether antitumor immunity is initiated, maintained, suppressed, excluded, or reshaped during therapy. The practical conceptual shift is to view checkpoint resistance as a lesion-level problem of immune access, competence, and suppression. We follow the clinical grouping used by major advanced gastric/GEJ adenocarcinoma trials, while explicitly separating gastric-derived evidence from GEJ-specific or combined gastric/GEJ validation.

### Applicability to gastroesophageal junction adenocarcinoma and site-specific evidence gaps

Although pivotal immunotherapy trials commonly group gastric and gastroesophageal junction adenocarcinoma, the spatial evidence base is uneven across anatomical sites. Most single-cell and spatial atlases discussed here derive from gastric cancer cohorts and include limited GEJ-specific annotation. GEJ adenocarcinoma may differ in epithelial lineage, tissue architecture, inflammatory background, stromal interface, lymphatic drainage, endoscopic sampling depth, prior treatment exposure, and metastatic pattern. For this reason, gastric cancer-derived spatial findings are treated as biologically informative hypotheses, not automatic GEJ selection criteria. Where possible, we indicate whether a feature is supported by gastric/GEJ ICI outcome-linked data, gastric spatial-biology or prognostic data, pan-cancer evidence, or a more exploratory inference.

### Methods: literature selection and evidence-tiering approach

This article is a narrative Review with structured evidence selection and evidence-tier annotation; it is not a systematic review, scoping review, or mapping review. Searches were last run on 26 May 2026 in PubMed, Web of Science, Google Scholar, ClinicalTrials.gov, and publisher websites using combinations of the terms gastric cancer, gastroesophageal junction cancer, immune checkpoint inhibitor, immunotherapy resistance, PD-1, PD-L1, single-cell RNA sequencing, spatial transcriptomics, spatial proteomics, multiplex immunofluorescence, tertiary lymphoid structure, cancer-associated fibroblast, macrophage, liver metastasis, peritoneal metastasis, and tumor microenvironment. The search was intended to support a structured narrative synthesis rather than exhaustive evidence capture. We prioritized peer-reviewed studies, pivotal clinical trials, gastric cancer-specific single-cell or spatial studies, and mechanistic or translational work that directly informs spatial immune resistance. Conference abstracts without adequate data, purely technical studies without cancer-relevant interpretation, and unpublished or inaccessible materials were excluded. Non-English articles were not systematically reviewed, which may underrepresent some regional evidence. Candidate studies were screened for relevance to the framework and assigned to one of four evidence tiers: gastric/GEJ ICI outcome-linked evidence, gastric prognostic or spatial-biology evidence, pan-cancer mechanistic or non-gastric ICI evidence, or hypothesis-generating concepts. Because the objective was conceptual synthesis rather than exhaustive enumeration, no PRISMA flow diagram, protocol registration, formal risk-of-bias scoring, or meta-analysis was performed.

### Contribution to the field

This Review takes immunotherapy resistance as the organizing clinical problem, rather than presenting a technology catalogue or biomarker inventory. Its main contribution is a three-axis framework based on immune accessibility, immune competence, and immune suppression that links tumor-immune-stromal architecture to clinically recognizable resistance phenotypes. The Review also maps immune-desert, immune-excluded, inflamed-dysfunctional, TLS-rich immune-active, and metastatic-niche states; separates outcome-linked evidence from prognostic or descriptive spatial biology; and outlines how spatial readouts might be developed as lesion-level explanatory biomarkers and pharmacodynamic endpoints. This focus aligns with the Frontiers in Oncology Gastrointestinal Cancers: Gastric and Esophageal Cancers section, particularly its interest in gastric/GEJ immune-stromal microenvironments, biomarkers, immuno-oncology, and single-cell or spatial multi-omics linked to treatment response or resistance.

## A spatial immune-ecology framework for interpreting gastric cancer immunotherapy resistance

Bulk transcriptomic and immunohistochemical analyses can distinguish immune-enriched from immune-poor gastric tumors, but they compress spatially heterogeneous tissues into averaged molecular or cellular signals (14, 15). A bulk interferon-gamma (IFNG) signature, for example, may arise from diffuse intratumoral T-cell infiltration, a small tertiary lymphoid structure (TLS)-rich region, inflamed mucosa adjacent to tumor, or a disorganized mixture of tumor and stromal compartments (16). High CD8A expression is likewise often read as evidence of an inflamed, immune-reactive phenotype, although its biological and clinical meaning depends on where CD8+ T cells reside (31). CD8+ T cells in direct contact with malignant epithelial cells may support effective cytotoxicity, whereas CD8+ T cells retained in peritumoral stroma by dense extracellular matrix, CAF-rich barriers, or abnormal vasculature may have limited opportunity to kill tumor cells (31–33). Spatial context therefore separates productive antitumor immunity from ineffective immune exclusion within tumors that can look similarly inflamed in bulk assays (16). Single-cell profiling resolves many of the participating cell states (18–22); spatial profiling determines how those states are assembled in tissue (27–30).

**Figure 1:**
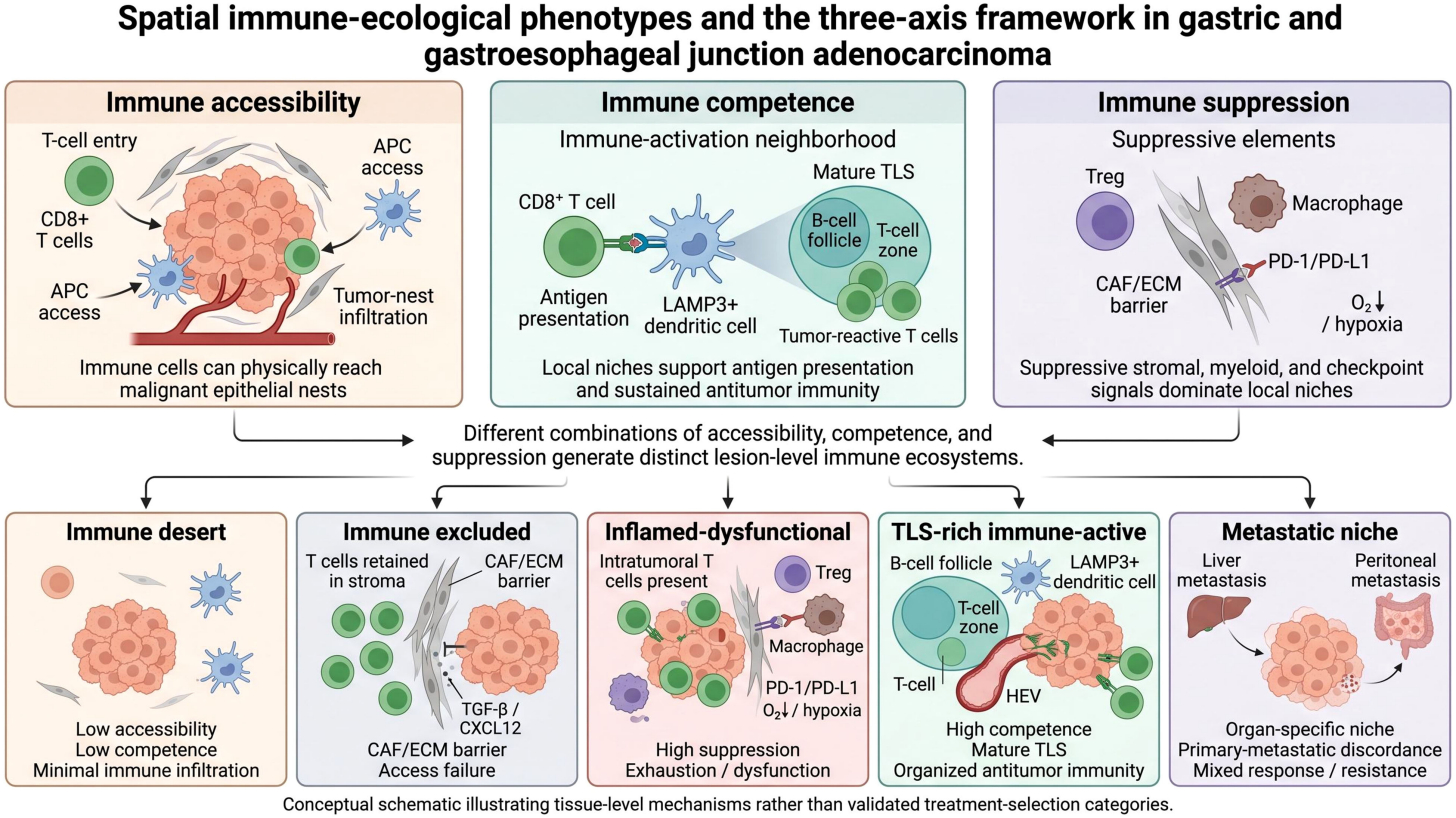
Spatial immune-ecological phenotypes and the three-axis framework. The schematic summarizes how immune accessibility, immune competence, and immune suppression combine to generate immune-desert, immune-excluded, inflamed-dysfunctional, TLS-rich immune-active, and metastatic-niche states. This schematic is conceptual and illustrates tissue-level mechanisms rather than validated treatment-selection categories.

To synthesize these interactions, we propose the integrative framework illustrated in **Figure 1**, which interprets gastric/GEJ immunotherapy response through three linked spatial axes: immune accessibility, immune competence, and immune suppression. Immune accessibility asks whether effector lymphocytes and antigen-presenting cells can cross vascular and stromal barriers and enter malignant epithelial nests. Immune competence refers to whether local neighborhoods contain the antigen-presentation machinery, T-cell clonality, progenitor-like exhausted T-cell pools, dendritic-cell maturation, and TLS architecture needed to sustain tumor-reactive immunity. Immune suppression captures the local dominance of regulatory T cells, suppressive myeloid populations, CAF-mediated ECM barriers, abnormal vasculature, hypoxia, metabolic stress, and inhibitory ligand networks. Different combinations of these axes generate recurring spatial immune-ecological phenotypes, from immune-desert and immune-excluded tumors to inflamed-dysfunctional, TLS-rich immune-active, and metastatic-niche states (23, 25, 39).

This framework also explains why a single tumor may contain sensitive and resistant microregions. A biopsy taken from a TLS-rich tumor edge can overstate global immune activation, while the deeper invasive front of the same lesion may be dominated by collagen-rich CAFs, suppressive macrophages, and physically excluded T cells (23, 29). Such neighborhood-level heterogeneity offers a tissue-level explanation for the clinical phenomenon of a mixed response (10, 11). A patient receiving PD-1 blockade, for example, may show regression of a primary gastric lesion while a liver metastasis or peritoneal implant progresses. Those divergent trajectories may partly reflect metastatic niches with immune architectures shaped by organ-specific stroma, distinct myeloid ontogeny, and unique vascular features, rather than simply the biology of the primary site (16, 22). Resistance is therefore better viewed as a dynamic, spatially defined phenotype that can vary within one lesion and across disseminated sites, not merely as a static cancer-cell-intrinsic molecular status (10, 11).

### Box 1. Evidence-tier language used in this review

- Gastric ICI outcome-linked evidence refers to gastric or gastroesophageal junction cohorts in which a spatial, single-cell, molecular, or histologic feature is directly associated with ICI response, progression-free survival, overall survival, or primary resistance during ICI-based therapy.
- Gastric prognostic or spatial-biology evidence refers to gastric cancer studies that link a feature to survival, clinicopathologic behavior, or tumor biology without directly linking it to ICI outcome. These findings should not be described as predictive unless treatment-linked validation is available.
- Pan-cancer mechanistic or non-gastric ICI evidence refers to experimental, cross-tumor, or other-cancer studies that support mechanisms such as T-cell exclusion, T-cell exhaustion, TLS biology, CAF-mediated barriers, myeloid suppression, or metabolic immune dysfunction. These data are most useful for generating gastric cancer hypotheses rather than gastric selection criteria.
- Hypothesis-generating evidence refers to computational signatures, ligand-receptor inferences, proposed composite scores, or candidate therapeutic combinations. Such concepts need orthogonal validation and prospective testing before clinical use.

For clarity, the spatial features discussed below should be read through this evidence ladder. Outcome-linked signals can shape clinical-trial stratification hypotheses; prognostic or descriptive spatial-biology findings nominate mechanisms; pan-cancer mechanisms require gastric/GEJ validation; and computational or composite scores remain exploratory until they are prospectively tested and reduced to clinically scalable assays.

## Clinical context and limitations of current biomarkers

The integration of PD-1 blockade into first-line therapy establishes immunotherapy as a major treatment modality in advanced gastric and gastroesophageal junction adenocarcinoma, although indications, biomarker thresholds, and approved regimens remain guideline- and region-dependent. CheckMate 649 demonstrated improved survival with nivolumab plus chemotherapy compared with chemotherapy alone, particularly in tumors with higher PD-L1 combined positive score (CPS) (4). KEYNOTE-859 supported pembrolizumab plus chemotherapy in HER2-negative disease, with efficacy evaluated across thresholds such as CPS ≥1 and ≥ 10 (5). ORIENT-16 and RATIONALE-305 further showed that PD-1 blockade combined with chemotherapy improves outcomes in large randomized settings; RATIONALE-305 used the Tumor Area Positivity (TAP) score (6, 7, 40). These assay-dependent scoring systems and operational cutoffs, including CPS ≥1, ≥5, and ≥10 and TAP-based thresholds, expose a practical limitation: numerical PD-L1 indices incompletely capture the spatial organization of immune activation, exclusion, and suppression.

The modern clinical landscape also includes biomarker-selected targeted therapy. In HER2-positive disease, trastuzumab-based therapy may be combined with PD-1 blockade and chemotherapy in appropriate clinical and regional settings, raising questions about how antibody-dependent immune mechanisms, macrophages, natural killer cells, and T-cell activation are arranged in physical space (9, 41). In HER2-negative, CLDN18.2-positive disease, SPOTLIGHT and GLOW support CLDN18.2-directed therapy as a clinically relevant first-line option for eligible patients, although implementation depends on regional approvals, assay availability, and guideline context (42, 43). CLDN18.2 is a tight-junction target rather than an immune checkpoint biomarker, but CLDN18.2-directed antibodies such as zolbetuximab are designed to induce tumor-cell killing through mechanisms that include antibody-dependent cellular cytotoxicity and complement-dependent cytotoxicity (44). These regimens define established or emerging clinical contexts; they do not yet create spatially selected treatment recommendations.

Clinical biomarkers remain essential, but their interpretation is sharpened by tissue context. PD-L1 CPS can be affected by assay platform, spatial sampling bias, tumor ulceration, and whether PD-L1 is expressed by malignant cells, macrophages, dendritic cells, or stromal cells. An aggregate CPS or TAP score cannot distinguish PD-L1-expressing immune cells engaged at a tumor nest from similar cells scattered in spatially separated fibrotic stroma (40). MSI-high/dMMR tumors are often inflamed and immunogenic, but antigenicity alone does not guarantee immune access. Antigen-presentation defects, CAF-driven stromal exclusion, or suppressive spatial niches such as localized danger zones may restrict CD8+ T cells despite hypermutated biology (45). EBV-positive tumors often show immune-rich features and checkpoint ligand expression, yet their organization may range from structured TLSs to diffuse dysfunctional inflammation. HER2 and CLDN18.2 expression can likewise vary substantially within a single lesion (46). Bulk biopsies can miss this subclonal spatial variation, producing lesion-level and regional differences in target accessibility and treatment sensitivity.

Spatial analysis can complement existing biomarkers in four concrete ways. It distinguishes productive immune infiltration within malignant epithelial regions from immune cells stranded in peritumoral stroma (31). It tests whether antigen-presenting dendritic cells are positioned close enough to T cells and tumor cells to support ongoing immune priming. It quantifies suppressive neighborhoods, including CAF-rich desmoplastic barriers, macrophage-dense invasive fronts, and localized hypoxic niches that constrain effector function (33, 38). Finally, it evaluates the maturity and anatomical position of TLS-like structures, clarifying whether they are integrated into the tumor bed, confined to the peritumoral margin, or disconnected from malignant regions (35, 36). These dimensions may help explain why tumors with similar conventional biomarker profiles follow different clinical trajectories.

**Table 1** summarizes how established and biologically informative gastric/GEJ biomarkers can be paired with spatial readouts. These readouts are not substitutes for guideline-supported testing; they are tissue-level refinements that may help explain discordant or mixed outcomes once assessed in outcome-linked cohorts and, where relevant, GEJ-annotated populations.

**Table 1:** Current gastric/GEJ biomarkers and proposed spatial complements.

Current biomarker	Clinical use or biological context	Key limitation	Spatial complement	Evidence boundary and site coverage
PD-L1 CPS or TAP score	Selects or enriches patients for PD-1-based treatment in advanced gastric/GEJ adenocarcinoma, depending on assay, regimen, and region.	Aggregate scoring lacks proximity information and is sensitive to assay, cutoff, ulceration, and sampling region.	Map PD-L1+ cells by compartment and test proximity to PD-1+ T cells within tumor nests, invasive margins, and macrophage-rich stroma.	Clinical scoring is outcome-linked in gastric/GEJ trials; spatial refinement remains investigational and requires site-specific validation.
MSI-high/dMMR	Identifies highly immunogenic tumors and supports ICI sensitivity in gastric/GEJ cancer.	High antigenicity does not guarantee antigen presentation, T-cell entry, or functional immune competence.	Assess intratumoral CD8+ infiltration, HLA/B2M status, dendritic-cell proximity, TLS maturity, and CAF/myeloid barriers.	Clinical value established; spatial modifiers require testing, especially in GEJ-annotated and metastatic cohorts.
EBV-positive status	Defines an immune-enriched molecular subgroup with frequent checkpoint-ligand expression.	Immune enrichment can range from organized TLS-rich immunity to diffuse dysfunctional inflammation.	Map TLS maturity, PD-L1 compartment, dendritic-cell/T-cell proximity, and background mucosal lymphoid inflammation.	Biologically informative; predictive relevance of spatial organization requires outcome-linked validation.
TMB-high context	May indicate neoantigen load and immune recognition depending on assay and threshold.	Thresholds and clinical utility are less standardized in gastric cancer than MSI/MMR status.	Combine antigenicity with spatial access, antigen-presentation integrity, and suppressive-neighborhood mapping.	Supportive or context-dependent biomarker; spatial access and antigen-presentation competence remain unvalidated modifiers.
HER2 expression or amplification	Guides HER2-targeted therapy and combinations with chemotherapy and PD-1 blockade in appropriate settings.	Spatial and lesion-level heterogeneity can produce regional target escape and divergent response.	Map HER2+ nests, effector-cell proximity, macrophage/NK-cell access, and post-treatment antigen-presentation changes.	Established clinical biomarker; spatial heterogeneity is a resistance hypothesis and pharmacodynamic readout, not a replacement assay.
CLDN18.2 expression	Guides CLDN18.2-directed antibody therapy in eligible HER2-negative disease, depending on approval and assay context.	Heterogeneous tight-junction expression may not reflect immune-cell access or antibody-effector engagement.	Map CLDN18.2+ tumor regions, stromal accessibility, complement or Fc-effector neighborhoods, and antigen release after therapy.	Clinically relevant target in eligible patients; spatial immune consequences and lesion-level heterogeneity still require validation.
Histology, primary site, and metastatic site	Supports clinical risk assessment and sampling decisions.	Diffuse-type disease, peritoneal implants, liver metastases, and GEJ tumors may differ from superficial primary biopsies.	Compare intestinal versus diffuse architecture, invasive front versus mucosa, and primary versus peritoneal, liver, nodal, or GEJ immune ecology.	Site and histology are essential annotation variables; GEJ-specific spatial validation remains limited.

## Emerging gastric ICI outcome-linked spatial evidence and candidate features

Direct gastric/GEJ ICI outcome-linked spatial evidence is still limited and should be kept distinct from prognostic or pan-cancer evidence. The clearest treatment-linked spatial signal discussed here comes from gastric cancer cohorts treated with anti-PD-1/PD-L1 plus antiangiogenesis, in which spatial transcriptomics and plasma proteomics identified microenvironmental features associated with primary resistance (116). These data support the clinical relevance of tissue-level spatial resistance. At the same time, the combination-therapy context, cohort size, and need for broader prospective testing mean that the identified features should not yet be used as independent selection criteria.

Gastric cancer spatial atlases of tumor progression, TLS organization, lymphocyte-aggregated regions, CCL2+ fibroblast-STAT3-activated macrophage neighborhoods, danger-zone fibroblast programs, HER2 heterogeneity, and the FMR1-FTO axis provide important spatial-biology, prognostic, or mechanistic evidence (23–26, 45, 46, 57). They nominate plausible resistance mechanisms but do not, on their own, establish predictive biomarkers for PD-1/PD-L1 blockade. A similar boundary applies to pan-cancer and non-gastric evidence supporting CXCL13+ T cells, TLSs, T-cell exhaustion states, CAF-mediated exclusion, myeloid suppression, and metabolic immune dysfunction (35, 36, 48, 79).

In that context, TLS maturity, CXCL13+ T-cell programs, LAMP3+ dendritic-cell proximity, CAF-myeloid neighborhoods, metabolic danger zones, and SICS-like composite spatial readouts are discussed as translational leads. The next task is not to name more spatial features, but to carry the most plausible ones from descriptive or prognostic atlases into gastric/GEJ ICI outcome-linked cohorts with prespecified endpoints, independent validation, and assays that can plausibly scale beyond discovery platforms.

### Single-cell atlases: defining the cellular cast of resistance

Single-cell transcriptomic atlases have supplied a cell-resolved inventory across premalignant, primary, and metastatic disease contexts, clarifying the cellular components that drive gastric cancer evolution and immune evasion (18–22). Across the developmental continuum, early scRNA-seq studies of premalignant lesions and early gastric cancer show that immunosuppressive niches, marked by stromal remodeling and early immune dysfunction, can arise before advanced disease is clinically apparent (18). In established primary gastric adenocarcinoma, malignant epithelial cells display deep transcriptional heterogeneity, adopting stem-like, proliferative, and lineage-specific programs that may influence antigenicity, stress responses, and immune-stromal interactions (19–21). In metastatic disease, particularly peritoneal carcinomatosis, single-cell profiling reveals extensive lineage diversity and organ-specific microenvironmental remodeling (22). For immunotherapy resistance, this continuum matters because the ecosystems that limit T-cell function are not static: they evolve from early local immunosuppression to complex, site-specific barriers in metastatic niches. Representative single-cell, immune-profiling, and spatial studies that inform this framework are summarized in **Supplementary Table 2**.

A major contribution of single-cell profiling is the shift from counting immune cells to interpreting their state and tissue position. A tumor may contain abundant CD8+ T cells yet resist therapy if those cells are terminally exhausted, metabolically impaired, or spatially disconnected from antigen-presenting niches. High PD-1 (PDCD1) expression does not automatically mean functional impotence. Rather, the balance between progenitor-like exhausted T cells, often marked by TCF7 and capable of proliferating after PD-1 blockade, and terminally exhausted subsets shapes the restorative potential of immunotherapy (34, 47). CXCL13+ CD8+ T cells often show clonal expansion, tumor-reactive features, and concurrent exhaustion programs; in gastric cancer, however, they remain response-associated candidates rather than validated standalone predictors until confirmed in ICI outcome-linked cohorts (48). Single-cell T-cell receptor (TCR) sequencing adds clonal context, but whether cytolytic potential is executed still depends on spatial access (49).

The CD4+ T-cell ecosystem in gastric cancer is similarly heterogeneous. Regulatory T cells (Tregs), characterized by FOXP3, IL2RA, and high expression of inhibitory receptors, accumulate in tumor and adjacent stromal niches and suppress immunity through CTLA4 engagement, IL-10 and TGF-β secretion, and local metabolic competition (19–22). By contrast, T follicular helper (Tfh)-like cells, marked by CXCL13, BCL6, and PDCD1, support B-cell maturation and contribute to the organization and maintenance of tertiary lymphoid structures (TLSs) (35, 36). Th1-like CD4+ populations expressing TBX21 and IFNG can cooperate with CD8+ T cells to reinforce local cytotoxicity. Because these CD4+ states may share activation markers while performing divergent functions, spatial transcriptomics is particularly useful for distinguishing a CD4+ T cell embedded within an immune-supportive TLS from one contributing to a suppressive peritumoral barrier (23–25, 27–31).

Given its mucosal origin and frequent association with chronic inflammation, Epstein-Barr virus (EBV), and Helicobacter pylori infection, gastric cancer often contains substantial B-cell and plasma-cell populations. Abundance alone, however, does not reliably signal favorable prognosis or intrinsic immunotherapy sensitivity (35, 50). Single-cell analyses reveal a heterogeneous B-cell compartment in which antigen-presenting B cells, germinal-center-like B cells, plasma cells, and regulatory B-cell states coexist. The meaning of this infiltrate becomes clearer only when it is linked to structure. Cross-tumor evidence, including renal cell carcinoma (51), together with emerging gastric spatial data (24, 25), suggests that B cells are most likely to support robust antitumor immunity when incorporated into mature TLSs with distinct T-cell zones, mature dendritic cells, and high endothelial venule-like structures (35, 36, 50, 51).

Myeloid cells form one of the most plastic axes of immunotherapy resistance. Tumor-associated macrophages (TAMs) span phagocytic, inflammatory, angiogenic, and lipid-associated programs (37). Across gastrointestinal tumors, spatial studies have implicated specific neighborhoods, including SPP1+ macrophage interactions with cancer-associated fibroblasts, in desmoplastic remodeling and T-cell exclusion (52). The dendritic-cell (DC) compartment in gastric cancer is similarly context dependent. Conventional DCs and LAMP3+ mature DCs can present antigen, but their functional effect depends on where they reside. LAMP3+ DCs within lymphoid aggregates may efficiently prime or restimulate T cells, whereas those held at fibrotic tumor margins may be constrained by suppressive signals (24, 25, 37). Neutrophils and monocyte-derived cells add further complexity by promoting localized hypoxia, extracellular-matrix remodeling, and angiogenesis. Thus, myeloid cells may initiate immunity or construct spatial barriers depending on the tissue niche they occupy (37, 38).

Fibroblasts, endothelial cells, and perivascular cells build the physical and metabolic scaffolding of immune resistance, although immune-focused biomarker studies have often underrepresented them. Single-cell profiling has moved beyond a monolithic view of cancer-associated fibroblasts, delineating myofibroblastic CAFs, inflammatory CAFs, antigen-presenting-like CAFs first characterized in pancreatic cancer models, perivascular states, and matrix-remodeling programs (53). This framework does not treat all stroma as suppressive. TGF-β-driven matrix-remodeling programs can exclude lymphocytes and prevent access to tumor nests (32, 54), whereas other stromal networks may restrain dissemination or support high endothelial venules and TLS formation (33). Effective intervention will likely require reprogramming selected suppressive stromal states while preserving tissue architecture that supports immunity (55). Across these atlases, the major gastric/GEJ-specific gap remains the scarcity of cohorts that combine treatment exposure, ICI outcomes, matched metastatic lesions, and spatial validation.

### Spatial multi-omics: mapping immune neighborhoods in intact tissue

Spatial transcriptomics and spatial proteomics provide the coordinate system that dissociation-based single-cell atlases cannot supply. Spot-based platforms capture genome-wide transcriptomes across broad tissue sections but usually aggregate several cells per spot, making them powerful for unbiased discovery of regional tumor programs. Higher-resolution *in situ* platforms and multiplex imaging methods, including IMC, mIF, CODEX, and MIBI, localize selected proteins or transcripts at single-cell or subcellular resolution, while ROI-based approaches such as GeoMx digital spatial profiling enable targeted or broad molecular profiling of anatomically defined tissue regions (27–30). The issue is therefore less a hierarchy of platforms than a match between platform and biological question: unbiased spatial grids are well suited to discovering microenvironmental domains, whereas targeted single-cell-resolution methods are better suited to testing proximity-based cellular interactions that can be prioritized for functional validation.

Recent gastric cancer spatial multi-omics studies illustrate both the value of this coordinate system and the need to respect evidence boundaries. Integrated spatial and single-cell profiling of gastric adenocarcinoma progression has linked intratumoral heterogeneity with distinct microenvironmental states and localized checkpoint expression (23). TLS-focused spatial transcriptomics has described immune-active TLS patterns enriched for CXCL13+ T lymphocytes, germinal-center B cells, LAMP3+CD80+ mature dendritic cells, and HEV-associated cells, with prognostic relevance in gastric cancer (24). Another spatial atlas identified dense lymphocyte-aggregated regions in which PD-1+CD27+CD8+ T cells lie close to CD70+LAMP3+ dendritic cells, suggesting a niche compatible with local immune activation (25). These architectures are strong markers of putative immune competence, but their conversion into predictors of PD-1 blockade will require prospective immunotherapy-linked cohorts and, where feasible, functional validation.

Spatial data move immuno-oncology beyond the binary question of whether immune cells are present. They ask whether effector T cells penetrate malignant nests or stop at stromal margins; whether exhausted T cells and mature dendritic cells are close enough to interact; whether B cells and T cells form mature TLSs rather than disorganized inflammatory aggregates; whether CAF-rich ECM or abnormal vessels separate lymphocytes from glands; whether PD-L1 is expressed where PD-1+ T cells reside; and whether metastatic lesions preserve or rewrite the immune architecture of the primary tumor (23, 31–39). Many gastric spatial atlases remain biology- or prognosis-oriented, and GEJ-specific outcome-linked spatial validation remains sparse.

## Ecological phenotypes of immunotherapy resistance

Spatial immune ecology frames gastric cancers as a spectrum of overlapping ecological phenotypes rather than rigid diagnostic categories (23, 39, 56–61). A single tumor can contain several phenotypes across different regions, and a metastatic lesion may diverge substantially from the primary tumor (22, 23, 46). Their translational value lies in linking tissue architecture to dominant resistance bottlenecks, sampling priorities, and testable clinical-trial hypotheses. The major spatial phenotypes, candidate biomarkers, temporal resistance patterns, and trial implications are summarized in **Table 2**.

**Table 2:** Spatial resistance phenotypes, temporal resistance classes, and sampling priorities.

Spatial phenotype	Dominant spatial scene	Likely resistance timing	Candidate spatial readouts and evidence level	Sampling or trial implication
Immune desert	Low T-cell and dendritic-cell infiltration in tumor and stroma; absent or immature TLSs.	Often primary resistance.	Candidate readouts: low intratumoral CD8, IFNG, GZMB, dendritic-cell recruitment, chemokine expression, and possible HLA/B2M defects. Evidence level: gastric/pan-cancer biology; ICI predictive value remains unproven.	Avoid overcalling from one biopsy; test priming strategies within biomarker-linked trials.
Immune excluded	T cells accumulate in stroma or margins but do not penetrate malignant nests because of CAF/ECM, TGF-beta, abnormal vessels, or myeloid barriers.	Primary or adaptive resistance.	Candidate readouts: high stromal but low tumor-nest CD8; CAF barrier thickness; CXCL12/TGF-beta activity; SPP1+ or STAT3-activated macrophages. Evidence level: gastric spatial biology/prognostic evidence with therapeutic rationale.	Sample invasive front; use on-treatment biopsies to test barrier remodeling.
Inflamed dysfunctional	T cells enter tumor regions but are constrained by exhaustion, Tregs, suppressive macrophages, inhibitory ligands, hypoxia, or metabolic stress.	Adaptive or acquired resistance.	Candidate readouts: PD-1/PD-L1 colocalization; CD8:Treg ratio; TCF7+ versus terminal exhaustion balance; IDO1/hypoxia/lactate markers. Evidence level: strong pan-cancer mechanism; gastric ICI outcome validation needed.	Use paired baseline and early on-treatment biopsies to measure reinvigoration and relief of suppression.
TLS-rich immune-active	Organized B-T-dendritic-cell niches with germinal-center features and high endothelial venule-like structures near tumor regions.	Sensitivity or adaptive resistance despite immune activity.	Candidate readouts: TLS maturity and tumor adjacency; CXCL13+ T cells; CXCR5/BCL6+ B cells; LAMP3+ DCs; HEV-like vessels. Evidence level: gastric prognostic/spatial biology; predictive value not established.	Distinguish mature intratumoral TLSs from background mucosal follicles; preserve productive TLSs in trial design.
Metastatic niche	Peritoneal, liver, nodal, or other organ-specific microenvironments diverge from the primary tumor in immune access, stroma, vasculature, and drug delivery.	Primary, mixed, or acquired resistance.	Candidate readouts: primary-metastatic discordance in PD-L1, HER2, CLDN18.2, immune architecture, CAF/myeloid dominance, hypoxia, and antigen-presentation status. Evidence level: strong biological rationale; gastric ICI outcome validation limited.	Biopsy progressing lesions when safe; build niche-specific pharmacodynamic endpoints.

The immune-desert phenotype is marked by sparse immune infiltration in both malignant tumor nests and surrounding stroma (14, 31). This immune-sparse state may reflect low tumor antigenicity, defective tumor-intrinsic antigen presentation, poor chemokine-mediated recruitment, exclusionary vascular networks, limited inflammatory priming, or absence of TLS-like organization (12, 16, 60). Such tumors typically show low PD-L1 expression and weak cytotoxic T-cell signatures, but the central failure is more fundamental: a tumor-reactive immune response is not generated, recruited, or retained in the relevant tissue compartment (12, 60). Spatial mapping is needed to distinguish a true biological immune desert from a false desert caused by sampling, in which a single-site core biopsy misses localized immune infiltrates elsewhere in the lesion (27, 40). For true immune-desert tumors, checkpoint blockade alone may be biologically insufficient; trial concepts may need to test priming strategies such as chemotherapy- or radiotherapy-induced immunogenic cell death, cancer vaccines, oncolytic viruses, STING or TLR agonists, or adoptive cellular therapies to initiate or amplify antitumor immunity (17, 60). These strategies remain exploratory in biomarker-defined gastric cancer cohorts.

The immune-excluded phenotype contains T cells and inflammatory signals but has a decisive spatial defect: lymphocytes accumulate in stroma or peritumoral margins and fail to penetrate malignant epithelial nests (14, 31, 60). In gastric cancer, this pattern is frequently linked to cancer-associated fibroblasts (CAFs), dense collagen deposition, robust TGF-β signaling, CXCL12 gradients, abnormal vasculature, and suppressive myeloid programs that construct a physical and biochemical barrier at the tumor-stroma interface (33, 57, 58). Classical pan-cancer studies identify TGF-β as an important mediator of stromal exclusion, redirecting T cells away from the tumor core (32, 62). This is a clear use case for spatial multi-omics: bulk RNA sequencing may label an immune-excluded tumor as highly inflamed because T-cell transcripts are abundant overall, whereas spatial assays can show that those T cells are trapped in the wrong compartment (15, 31, 63). The inflamed-dysfunctional phenotype contains abundant T cells within or immediately adjacent to tumor nests, yet the local microenvironment is dominated by exhaustion, Tregs, suppressive myeloid cells, inhibitory ligand networks, metabolic stress, and insufficient continuous antigen presentation (16, 38, 60). These tumors often express multiple co-inhibitory receptors, including PD-1, PD-L1, CTLA-4, LAG-3, TIGIT, TIM-3, and IDO1, together with interferon-stimulated gene signatures (16, 64). A highly inflamed signature, however, does not guarantee response to PD-1 blockade. Treatment efficacy depends on the functional state of the T cells: responses are often associated with expansion of a progenitor-like exhausted pool, whereas tumors dominated by terminally exhausted T cells may remain less responsive (34, 47, 65). Even when progenitor T cells are present, localized resistance can persist if they are surrounded by suppressive macrophages, high Treg density, or disrupted antigen-presenting-cell networks that fail to sustain reinvigoration (37, 38, 65). The TLS-rich or lymphocyte-aggregated phenotype is distinguished by organized assemblies of B cells, T cells, follicular helper-like programs, mature dendritic cells, and often high endothelial venules (HEVs) (35, 36, 66). When fully functional, TLSs can serve as local hubs for antigen presentation, T-cell priming, and B-cell maturation within the tumor microenvironment (24, 67). Yet not every lymphoid aggregate carries the same meaning. Recent spatial studies emphasize maturation status, including germinal-center organization, proximity to malignant regions, and cellular composition involving mature LAMP3+ DCs and Tfh cells (24, 25). Immature or distant clusters may reflect background chronic inflammation rather than effective antitumor immunity. Mature, structurally organized, tumor-adjacent TLS patterns should therefore be presented as markers of an immune-active architecture under investigation, not as established predictors of ICI response (24, 66, 67).

The metastatic-niche phenotype deserves separate attention because metastatic lesions may diverge from the primary tumor in immune access, stromal structure, vascular organization, drug delivery, and antigenic composition (22, 46, 59, 68). Peritoneal and liver metastases are especially relevant in gastric cancer. Both sites can generate organ-specific suppressive niches in which myeloid cells, fibroblasts, endothelial or mesothelial cells, and metabolic constraints reshape T-cell function. Matched primary-metastatic spatial profiling is therefore central to explaining mixed responses and lesion-level resistance during immunotherapy.

## Metastatic niches: peritoneal and liver ecosystems

Peritoneal metastasis is a dominant and clinically formidable route of dissemination in gastric cancer (22, 68). The peritoneal cavity contains mesothelial cells, specialized fibroblasts, ascites-associated suppressive macrophages, adipocyte-derived metabolic signals, and mechanical fluid shear stress (22, 59, 68). This niche can impose poor physical access for T cells, suppressive myeloid metabolism, abnormal fibroblast-mesothelial crosstalk, and altered drug delivery dynamics, helping to explain why peritoneal implants may progress even when immune activity is detectable in the primary lesion (59, 68, 101).

Liver metastasis represents another organ-specific immune ecosystem. The liver is intrinsically biased toward immune tolerance through liver sinusoidal endothelial cells, Kupffer cells, recruited monocyte-derived macrophages, stellate cells, and specialized vascular architecture. In gastric cancer liver metastasis, emerging molecular pathology and spatial-translational studies suggest that metastatic colonization is accompanied by immune, stromal, and metabolic remodeling distinct from both the primary stomach lesion and other metastatic sites (111, 112). Most available liver-metastasis data, however, remain spatial-biology or review-level evidence rather than gastric ICI outcome-linked validation.

For clinical trials, metastatic-site sampling should be treated as part of the biological question rather than a logistical detail. When safe and feasible, a progressing peritoneal or liver lesion may be more informative than a responding primary tumor. Paired primary-metastatic analysis can test whether resistance is driven by antigen loss, local stromal exclusion, myeloid dominance, hypoxic metabolism, spatial heterogeneity of HER2 or CLDN18.2, or organ-specific drug delivery barriers.

## Tumor-intrinsic programs and spatial immune escape

Tumor cells actively shape their local immune ecology through antigenicity, interferon signaling, chemokine expression, stress responses, epithelial-mesenchymal plasticity (EMT), metabolic programs, and lineage states (10, 11, 13). MSI-high and Epstein-Barr virus (EBV)-positive gastric cancers tend to be immunogenic because of elevated neoantigen loads or viral antigens, but spatial organization determines whether this potential is realized in tissue (12, 16). Antigen-rich regions may coexist with poorly antigenic or immune-edited subclones, creating regional therapeutic blind spots (13). Beyond antigenicity, tumor-intrinsic genetic programs can actively sculpt immune exclusion. Established pan-cancer paradigms, increasingly relevant to gastrointestinal tumors, show that allele-specific HLA loss (69) or inactivating B2M mutations impair antigen presentation, whereas acquired JAK1 or JAK2 mutations disrupt IFN-γ signaling and reduce responsiveness to T-cell-derived effector cytokines (70). Intrinsic oncogenic networks, including WNT/β-catenin signaling, can further promote immune exclusion by impairing dendritic-cell recruitment (71, 72). In gastric cancer, EMT- and stemness-associated programs can correlate with stromal desmoplasia, physically barricading tumor nests from immune infiltration and contributing to adaptive or acquired resistance (73).

Importantly, these tumor-intrinsic antigen-presentation defects are spatially consequential because immune accessibility does not necessarily equal immune competence. Tumor regions with HLA class I downregulation, HLA loss of heterozygosity, B2M alteration, or defective antigen-processing machinery may be physically infiltrated by CD8+ T cells but remain functionally invisible to cognate cytotoxic recognition (69, 70). This creates a state of spatial access without functional recognition, in which T cells are located within or near malignant epithelial nests but fail to execute effective killing. In spatial maps, such regions may appear immune-infiltrated yet behave as locally resistant microdomains, especially when tumor-cell contact is not accompanied by intact HLA/B2M expression, interferon responsiveness, dendritic-cell support, or clonal evidence of tumor reactivity (69, 70, 81, 82).

Malignant epithelial states identified by single-cell analysis occupy different tissue niches, a point especially relevant to gastric cancer’s histologic diversity (21, 23). Under the Lauren classification, intestinal-type gastric cancers often form glandular structures that can support localized immune infiltration or margin-based exclusion. Diffuse-type gastric cancers, particularly signet-ring cell tumors aligned with the Genomically Stable (GS) molecular subtype, are characterized by frequent E-cadherin (CDH1) dysfunction or loss (2, 3). These diffuse malignant programs infiltrate the gastric wall and can organize a desmoplastic, therapy-resistant microenvironment through chemokines, growth factors, and extracellular-matrix (ECM)-interacting molecules (74). Spatial transcriptomics is now positioned to define how these lineage states shape immune suppression in the hypoxic tumor core, invasive front, mucosal surface, and disseminated metastatic implants (23, 29).

PD-L1 expression is itself spatial. It can be induced dynamically by local IFN-γ released by invading T cells, producing adaptive immune resistance in inflamed tumor neighborhoods (75). It may also be driven constitutively by oncogenic pathways or viral mechanisms, or expressed mainly by stromal and immune cells, including tumor-associated macrophages, rather than malignant epithelium (38, 75). This distinction exposes a limitation of the PD-L1 combined positive score (CPS) used clinically (4, 5, 40). Because CPS blends tumor-cell and immune-cell PD-L1 positivity into one aggregate index, it removes proximity and neighborhood information (40, 75). PD-L1-positive macrophages sequestered in dense fibrotic stroma far from tumor nests may contribute to CPS without reflecting tumor-directed immune engagement (57). Spatial assays can resolve this ambiguity by distinguishing macrophage-rich PD-L1-positive zones from PD-L1-positive tumor nests and by testing whether PD-L1 is physically close to PD-1+ T cells where inhibitory signaling could occur (27–30, 40, 75). Taken together, these tumor-intrinsic programs make malignant epithelial cells active organizers of spatial immune escape, shaping whether a region evolves toward immune desertification, stromal exclusion, inflamed dysfunction, or metastatic-niche adaptation (10, 39).

## T-cell states, exhaustion, and tumor reactivity in space

T cells should not be interpreted by bulk abundance alone (49). Naive-like, central memory, effector memory, tissue-resident memory, progenitor exhausted, terminally exhausted, regulatory, follicular helper-like, and cytotoxic states may coexist within the gastric tumor microenvironment (21, 34, 49). PD-1 expression alone does not define terminal dysfunction; depending on co-expressed markers and location, it may mark transient activation, tumor reactivity, progressive exhaustion, or state transition (47, 64). TCF7+ progenitor exhausted cells positioned near antigen-presenting niches suggest a rescuable T-cell pool capable of proliferating after PD-1 blockade (34, 76, 77). Terminally exhausted T cells embedded in hypoxic, suppressive regions may be less amenable to reinvigoration (16, 65). The potential for checkpoint response therefore emerges from three interdependent dimensions: T-cell state, including progenitor versus terminal exhaustion; clonality, reflecting tumor-reactive expansion; and spatial location, reflecting access to antigen-presenting cells and malignant cells (76, 77).

CXCL13+ T cells are particularly relevant because they often represent tumor-reactive or chronically stimulated populations and are associated with TLS-like organization (48, 78). Pan-cancer analyses and emerging gastric spatial studies point to CXCL13 expression, TLS cellular composition, and dendritic cell-T cell proximity as response-associated or biologically plausible features (24, 25, 48). Still, CXCL13 expression is not uniformly favorable and should not be treated as a standalone biomarker. Its meaning depends on tumor-reactive clonality, integration with mature dendritic cells and TLSs, and whether CXCL13+ cells are located near or within tumor nests (48, 78). Spatial mapping is needed to determine whether these cells are embedded in malignant epithelial nests, organized within B-cell follicles, or confined to ineffective peritumoral inflammatory stroma (27, 79).

Regulatory T cells (Tregs) suppress antitumor immunity through CTLA-4 engagement, IL-10 and TGF-β secretion, metabolic competition, and direct interactions with antigen-presenting cells (19, 49, 62, 65). Their effect is strongly proximity-dependent (79). A high Treg density immediately adjacent to cytotoxic T cells and LAMP3+ dendritic cells within tumor nests is likely to impose greater local suppression than the same number of Tregs dispersed across distant, uninvolved mucosa (57, 79). This spatial dependency supports neighborhood-derived metrics, such as the CD8+ T-cell to FOXP3+ Treg ratio within a specified microscopic radius or the nearest-neighbor distance between Tregs and mature dendritic cells, as potentially more informative than bulk immune-cell counts (79). However, standardized distance cutoffs and cross-platform consistency have not been established for routine clinical implementation (80).

Combining single-cell T-cell receptor sequencing (scTCR-seq) with spatial transcriptomics or multiplex imaging links T-cell identity, clonal expansion, exhaustion trajectory, and physical position (29, 49). Expanded clones within tumor nests or mature TLS-like structures support active, antigen-driven immunity (49). By contrast, heavily expanded clones trapped in fibrotic stroma may indicate incomplete tissue access or failed effector deployment (31, 81). scTCR-seq remains biologically powerful but limited: clonal expansion does not prove tumor-antigen specificity (81). Many expanded intratumoral clones are virus-specific bystander lymphocytes without direct antitumor capacity (81). Inferring genuine tumor reactivity therefore requires integration with direct tumor-antigen validation, functional cytotoxicity assays, and/or spatial evidence of close contact with malignant epithelium, ideally supported by functional validation (81, 82). Accordingly, spatial proximity should be interpreted together with tumor antigen-presentation integrity. A cluster of CD8+ T cells near tumor cells may represent productive tumor recognition only when the malignant region preserves the relevant antigen-presenting machinery; otherwise, infiltrating or clonally expanded T cells may reflect inflammation-associated or virus-specific bystander activity rather than effective antitumor immunity (69, 81, 82).

## Myeloid niches: macrophages, dendritic cells, and neutrophils

Myeloid cells are highly plastic and can support or suppress antitumor immunity and immunotherapy response (37). Conventional type 1 (cDC1) and type 2 (cDC2) dendritic cells are central to cross-presentation to CD8+ T cells and CD4+ T-cell priming, respectively (83). Mature LAMP3+ dendritic cells, frequently corresponding to the mature regulatory DC (mregDC) program, have a more context-dependent biology (83). Rather than being uniformly immunostimulatory, LAMP3+ DCs express maturation and migratory markers such as CD80, CD86, and CCR7 together with immunoregulatory molecules including PD-L1 and IDO1 (83). In gastric cancer spatial studies, proximity between PD-1+CD27+CD8+ T cells and CD70+LAMP3+ dendritic cells suggests a potentially productive immune neighborhood (25). Such niches may support local restimulation of exhausted T cells after PD-1 blockade, but the function of LAMP3+ DCs depends heavily on whether they are located in lymphoid aggregates or suppressive stromal margins (25, 83).

Macrophage biology is too plastic for the traditional M1/M2 dichotomy to capture human tumor microenvironments (37, 84). Single-cell transcriptomics has redefined tumor-associated macrophages (TAMs) into functional programs, including SPP1-high states associated with angiogenesis, matrix remodeling, and cancer-associated fibroblast crosstalk; APOE-high or lipid-associated states linked to immunosuppressive programs; and complement-associated inflammatory states (37, 84). These programs occupy different spatial territories. SPP1+ macrophage-fibroblast neighborhoods and CCL2+ fibroblast-STAT3-activated macrophage crosstalk, for example, have been implicated in stromal infiltration and immunosuppressive spatial remodeling in gastric cancer (57, 84). Macrophage-rich invasive margins and hypoxic zones can sequester T cells and prevent effective attack on malignant epithelial nests (38, 57, 84).

Neutrophils and granulocytic myeloid-derived suppressor cells (PMN-MDSCs) contribute to tumor-associated inflammation, tissue injury, immune suppression, and metastatic progression (85). Their abundance and transcriptional diversity are often underestimated in routine scRNA-seq because of low cellular RNA content, high endogenous RNase activity, and fragility during tissue dissociation (85). Spatial proteomics, CyTOF, and optimized single-cell or single-nucleus workflows may therefore be needed to characterize them more completely (85). Spatially, neutrophils often localize near necrotic cores, abnormal vasculature, mucosal injury sites, and the tumor-stroma border (85). Within these niches, reactive oxygen species (ROS), matrix metalloproteinases, and neutrophil extracellular traps (NETs) can drive local immunosuppression and facilitate metastatic colonization (85).

Therapeutically, this myeloid complexity supports combination strategies beyond PD-1 monotherapy, but only as hypotheses matched to neighborhood biology (38, 86). Depending on the dominant niche, relevant interventions may include CSF1R pathway modulation, CCR2/CCR5 targeting to limit monocyte recruitment, CXCR2 inhibition against neutrophils, VEGF/VEGFR blockade, CD47-SIRPα disruption to enhance phagocytosis, or STING or TLR agonists to promote local dendritic-cell activation (38, 86). These approaches remain to be validated biomarker-guided strategies in gastric cancer. Their success will likely depend on spatial biomarkers that confirm not merely the presence of a target myeloid population, but its location in the compartment where it interacts with T cells or fibroblasts (38, 86). Myeloid-targeted therapy should therefore be guided by neighborhood function, not by macrophage, DC, or neutrophil abundance alone.

## Fibroblasts, extracellular matrix, and vascular barriers

Cancer-associated fibroblasts (CAFs) are central structural and biochemical organizers of immune exclusion in gastric cancer (33, 58). They produce and remodel extracellular matrix (ECM), secrete immunomodulatory chemokines and growth factors, regulate transforming growth factor-beta (TGF-β) signaling, and influence local vascular architecture (33, 54). Single-cell data resolve this historically monolithic population into operational states, including myofibroblastic CAFs (myCAFs, often characterized by ACTA2, COL1A1, and FAP expression), inflammatory CAFs (iCAFs, often marked by IL6 and CXCL12 secretion), antigen-presenting-like CAFs (apCAFs), perivascular states, and matrix-remodeling states (53, 55). These surface markers and secretory proteins should be read as dynamic programs rather than fixed identities. In spatial data, CAF subsets form organized barriers at invasive margins, perivascular tracks that govern leukocyte trafficking, or fibrotic stromal islands that separate T cells from malignant epithelial nests (57, 58).

Altered ECM controls T-cell motility through both physical and biochemical mechanisms. Dense collagen networks, fibronectin, hyaluronan, and highly aligned fibers can act as barricades that restrict perpendicular lymphocyte entry into tumor nests (33, 54). CAF-derived CXCL12 gradients, TGF-β, IL-6, macrophage migration inhibitory factor (MIF)-related signaling, and collagen-integrin interactions can redirect T cells, support suppressive myeloid recruitment, and maintain immune exclusion (32, 54, 57). A recent integrative single-cell, spatial, and bulk transcriptomic study in gastric cancer linked CAF-centered signaling networks and the FMR1-FTO epigenetic axis to an immune-excluded phenotype (26). This finding illustrates how spatially informed integration can nominate resistance pathways, but the FMR1-FTO axis remains a newly described candidate mechanism that requires functional and clinical validation before being considered a therapeutic target.

Vascular organization is equally important because even a primed immune response requires lymphocyte entry to execute cytotoxicity. Abnormal, leaky tumor vasculature can limit immune trafficking. Pan-cancer studies indicate that vascular endothelial growth factor (VEGF)-driven angiogenesis can promote endothelial anergy, impair dendritic-cell maturation, recruit regulatory T cells, and skew myeloid cells toward immunosuppressive states (16, 75). In contrast, high endothelial venule (HEV)-like structures, often embedded within mature TLSs, can support localized lymphocyte recruitment and sustain spatial immune organization (35, 36, 67). The coexistence of abnormal vasculature, hypoxia, desmoplastic CAFs, suppressive macrophages, and excluded T cells provides a mechanistic rationale for combining PD-1 blockade with anti-angiogenic or stroma-modulating strategies (16). In previously treated advanced gastric cancer, early-phase studies combining PD-1 inhibitors, including nivolumab or pembrolizumab, with the VEGFR2 antagonist ramucirumab have shown promising preliminary efficacy and manageable safety, consistent with the broader vascular-immune rationale (87).

Stromal targeting, however, requires restraint. Fibroblasts are highly heterogeneous, and selected populations restrain tumor growth, maintain tissue integrity, and structurally support TLS organization (55, 58). Pancreatic cancer preclinical evidence shows that broad, non-selective CAF depletion can be harmful in some models, promoting aggressive dissemination, increased hypoxia, and intensified immunosuppression (88). The more plausible goal is selective reprogramming of suppressive stromal states rather than widespread stromal ablation (55). Spatial multi-omics can help identify which fibroblast states create active immune barriers, which ligand-receptor pathways sustain exclusion, and which stromal features correlate with resistance or response to immunotherapy (23, 57).

## B cells, tertiary lymphoid structures, and lymphocyte-aggregated regions

Tertiary lymphoid structures (TLSs) are organized ectopic lymphoid aggregates that arise in chronically inflamed tissues and tumor microenvironments (89, 90). Their structural maturity needs explicit definition so that mature TLSs are not conflated with unorganized lymphocyte aggregates or diffuse mucosal inflammation (89). Mature TLSs show anatomical compartmentalization, typically including B-cell follicles with germinal-center programs, adjacent T-cell zones, follicular dendritic cell (FDC)-like networks, antibody-producing plasma cells, T follicular helper (Tfh)-like cells, mature dendritic cells, and specialized high endothelial venule (HEV)-like vessels (35, 36, 89). In several malignancies, such mature B-cell-rich immune contexts have been associated with prolonged survival and favorable responses to immune checkpoint blockade (35, 36, 50).

Gastric cancer is a particularly complex model for TLS research because chronic inflammation, Helicobacter pylori infection, and Epstein-Barr virus (EBV) frequently shape the background mucosal immune landscape (90). That background requires careful spatial separation: pre-existing mucosal lymphoid follicles or lymphocyte aggregates associated with chronic H. pylori gastritis should not be equated with tumor-associated *de novo* TLSs that may participate in organized antitumor immunity (90). The meaning of lymphoid structures depends on maturity, cellular composition, and location. A recent spatial transcriptomics study in gastric cancer, for example, stratified TLS patterns and reported that immune-active TLSs were enriched for CXCL13+ T lymphocytes, CXCR5+ germinal-center B cells, LAMP3+CD80+ activated dendritic cells, and HEV-associated endothelial cells (24).

A complementary spatial atlas identified dense lymphocyte-aggregated regions in which PD-1+CD27+CD8+ T cells were close to CD70+LAMP3+ dendritic cells (25). These observations suggest that organized lymphoid neighborhoods may act as structural hubs for local antitumor immunity, while also underscoring unresolved questions. TLSs should be evaluated across maturation, location, and density (89). Do resistant tumors lack TLSs entirely, or do they contain immature TLS-like aggregates without functional germinal centers? Are these structures intratumoral and close enough to malignant regions to support direct immune attack, or are they confined to distant peritumoral stroma? Do specific B-cell states, including IL-10-secreting regulatory B cells (Bregs), exert local immunosuppressive effects rather than promoting cytotoxic responses (91)? These distinctions will require multiplex immunofluorescence and spatial omics to define maturation state, cellular interactions, and coordinates beyond what standard histology can resolve (89).

Clinically, spatially defined TLS-related features may complement PD-L1 CPS and MSI status (89). A tumor-adjacent, structurally organized TLS-rich tumor with high CXCL13 expression and LAMP3+ dendritic-cell enrichment may indicate local immune organization and potential reactivity even when overall PD-L1 expression is modest. Diffuse B-cell infiltration without organized T-cell-dendritic-cell interactions is unlikely to carry the same meaning (24, 91). TLS presence should therefore be interpreted as a candidate marker of immune organization rather than a clinical mandate for PD-1 blockade. Future translational studies should distinguish mature intratumoral TLSs, peritumoral lymphoid aggregates, and background mucosal lymphoid inflammation in outcome-linked cohorts.

## Metabolic, microbial, and inflammatory gradients

This section is more exploratory than outcome-linked, because direct spatial evidence connecting metabolic or microbial gradients to ICI efficacy in gastric cancer remains limited. The stomach is a specialized mucosal organ exposed to gastric acid, dietary antigens, distinct microbial communities, and chronic tissue injury (92). The cascade from Helicobacter pylori-induced chronic active gastritis to atrophic gastritis, intestinal metaplasia, dysplasia, and carcinoma creates a spatially complex background of mucosal remodeling (92). Epstein-Barr virus (EBV)-positive tumors often show immune-rich features and checkpoint ligand expression, although the spatial organization of these responses varies (12, 16). These etiologic factors create inflammatory gradients across the mucosal surface, invasive front, necrotic regions, and adjacent non-neoplastic tissue, complicating interpretation of tumor-specific immunity (92).

Metabolic gradients may further shape localized immune dysfunction (93). In hypoxic tumor cores and poorly perfused desmoplastic stroma, hypoxia-inducible programs can promote VEGF production, adenosine pathway activation, glycolytic competition, suppressive macrophage states, lactate accumulation, and nutrient restriction (38, 93). Necrotic regions and dense invasive margins may also contain IDO1 activity, reactive oxygen species, and impaired T-cell or NK-cell cytotoxicity (93). The key spatial issue is whether these suppressive conditions occupy the same microscopic neighborhoods in which cytotoxic cells attempt to engage malignant cells. This is especially relevant for peritoneal ascites, where lipid-rich and nutrient-restricted environments may impair immune effector function (59, 93).

Microbial and viral gradients are an emerging frontier in gastric cancer spatial immune ecology (94). H. pylori-driven gastritis can generate dense lymphoid aggregates and strong inflammatory signatures in adjacent mucosa that may be mistaken for tumor-specific antitumor immunity in bulk analyses unless spatial context is considered (90, 92). Direct evidence linking precise microbial spatial localization to immune checkpoint blockade efficacy in gastric cancer remains limited, but broader microbiome-immunotherapy studies and spatial intratumoral microbiota analyses make this a priority area (94–96). Integrating spatial host transcriptomics with highly multiplexed microbial mapping and immune profiling may clarify whether particular intratumoral microbial niches recruit suppressive myeloid populations, alter checkpoint ligand expression, or contribute to localized immunotherapy resistance (94).

## Controversies and unresolved questions

Several controversies need to be addressed before spatial features can move toward clinical use. First, TLSs are not uniformly favorable. Mature, tumor-adjacent TLSs with germinal-center programs, Tfh-like cells, LAMP3+ dendritic cells, and HEV-like vessels may indicate immune competence, whereas immature, distant, or inflammation-associated aggregates may reflect background mucosal immunity rather than tumor-directed immune organization (24, 25, 89, 90).

Second, CXCL13+ T cells and expanded T-cell clones are not automatically predictive biomarkers. CXCL13 expression may mark tumor-reactive or chronically stimulated T cells, but it may also coexist with terminal exhaustion or bystander responses. Similarly, scTCR-seq demonstrates clonal expansion but does not prove tumor-antigen specificity. Spatial proximity to malignant epithelium, integration with mature dendritic-cell or TLS niches, and orthogonal functional validation are needed before these readouts can guide treatment (48, 49, 81, 82).

Third, stromal targeting is biologically attractive but clinically risky. CAF-rich and collagen-rich regions can exclude lymphocytes, yet selected fibroblast populations can restrain tumor growth, maintain tissue integrity, and support lymphoid architecture (33, 55, 58). Spatial biomarkers should identify suppressive barrier programs and relevant ligand-receptor circuits, not justify broad stromal ablation.

Fourth, GEJ extrapolation and composite spatial cutoffs remain unresolved. Most spatial evidence is gastric rather than GEJ-specific, and no standardized distance thresholds, compartment definitions, or cross-platform cutoffs have been accepted for routine clinical use. Studies claiming predictive utility should report site, histology, biopsy depth, region annotation, treatment exposure, assay platform, segmentation strategy, and clinical endpoint (97, 99, 109, 110).

## Toward composite spatial biomarkers

For clinical utility, a spatial biomarker has to move beyond descriptive biology and become an interpretable, reproducible, tissue-efficient metric linked to a decision or pharmacodynamic question. That requires translating cell abundance into measurable spatial parameters. Examples include compartment-specific density, such as CD8+ T cells per square millimeter within malignant epithelial nests versus stroma; compartment ratios, such as the CD8+ T-cell to FOXP3+ Treg ratio within tumor-infiltrating regions; and proximity metrics, such as nearest-neighbor distance between exhausted T cells and LAMP3+ dendritic cells or malignant cells (24, 25, 79). Other measurable parameters include TLS maturity and location (24, 25), CAF-rich exclusion-barrier thickness, enrichment of suppressive macrophage neighborhoods (57), and PD-1+/PD-L1+ spatial co-localization (79). Continuous distance or proximity metrics may capture immune engagement more directly than whole-slide bulk counts (24, 25, 57, 79, 97).

Because immunotherapy resistance is multifactorial, composite spatial frameworks may ultimately outperform single-feature assays (97). As a conceptual prototype rather than a validated clinical score, a SICS-like spatial immune-competence framework would integrate intratumoral CD8+ infiltration, CXCL13+ tumor-reactive T-cell programs, mature TLS proximity, and LAMP3+ dendritic-cell neighborhoods while accounting for stromal barrier metrics, TGF-β/CAF activity, SPP1-high macrophages, hypoxia, abnormal vasculature, and diminished antigen presentation. No cutoff should be proposed without validation. Clinical utility would require retrospective training in heavily annotated cohorts, independent external validation in multi-institutional datasets, and prospective testing against survival, response, and pharmacodynamic endpoints in randomized PD-1-based trials (97).

### Box 2. Minimum validation requirements before clinical use

- Compartment-specific immune infiltration: CD8+ T-cell density should be reported separately in malignant epithelial nests, invasive margins, and stroma rather than as a whole-slide average.
- Proximity-based immune competence: LAMP3+ or mature dendritic-cell proximity to PD-1+ or TCF7+/CXCL13+ T cells should be measured with prespecified distance rules and tested across platforms.
- TLS maturity and location: TLSs should be distinguished from unstructured lymphoid aggregates and background mucosal follicles using germinal-center markers, HEV-like vessels, cellular composition, and tumor adjacency.
- Suppressive barrier metrics: CAF/ECM thickness, macrophage-rich neighborhoods, hypoxic zones, and PD-1/PD-L1 co-localization should be linked to ICI endpoints rather than inferred from prognosis alone.
- Site-specific validation: gastric, GEJ, primary, peritoneal, liver, and nodal lesions should be annotated separately whenever possible, with independent external validation before clinical implementation.

**Figure 2:**
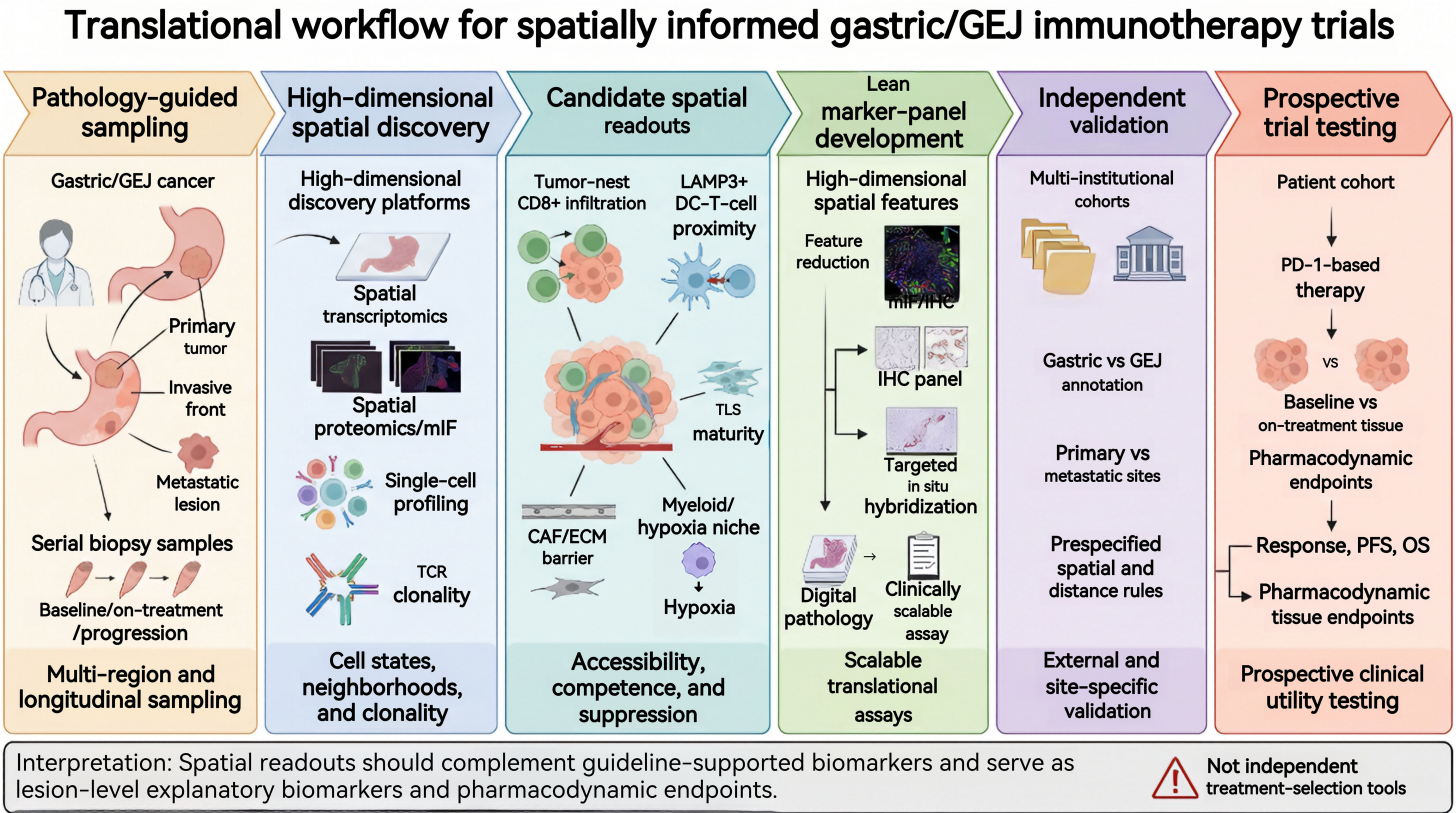
Translational workflow for spatially informed gastric/GEJ immunotherapy trials. The workflow moves from pathology-guided sampling and high-dimensional discovery to lean marker-panel development, independent validation, and prospective testing. Spatial readouts are framed as lesion-level explanatory biomarkers and pharmacodynamic endpoints rather than independent clinical selection tools.

Clinical implementation will require simplification. Comprehensive spatial transcriptomics and proteomics are invaluable for discovery but remain costly, time-consuming, and technically demanding for routine care (27–30). A plausible translational path begins with high-dimensional single-cell and spatial discovery, then reduces informative features to robust protein or transcript panels suitable for multiplex immunofluorescence, digital pathology-assisted immunohistochemistry, targeted *in situ* hybridization, or region-of-interest profiling (97). AI-based histopathology may help extract spatial immune features from routine H&E or single-stain IHC slides, but such models must be biologically anchored to validated molecular features and externally validated across scanners, staining protocols, and institutions to avoid overfitting (80, 98). **Figure 2** summarizes this translational workflow.

Spatial tissue sampling remains a central unresolved challenge. Routine endoscopic biopsies are superficial and may miss the deep invasive front, stroma-embedded TLSs, and metastatic immune niches. Multi-site endoscopic sampling, pathology-guided region selection, and metastatic-lesion biopsy should be considered when safe and ethically justified. Resection specimens provide broader tissue maps but often represent localized, treatment-naive disease rather than advanced, heavily pretreated immune ecology. Longitudinal baseline, early on-treatment, mixed-response, and progression biopsies would best define resistance evolution, although feasibility and patient burden are substantial. Spatial biomarker studies should transparently report tissue region, assay platform and resolution, segmentation algorithm, cell-type annotation, prior therapy exposure, and clinical endpoint (99).

Emerging biopsy approaches and liquid biopsy may partly address the longitudinal and systemic dimensions of this sampling problem. ctDNA, circulating tumor cells, and extracellular vesicle-based assays can provide minimally invasive, repeatable information on clonal evolution, molecular residual disease, acquired resistance, and lesion-level biomarker discordance when repeated deep tissue sampling is impractical (118, 119). This is particularly relevant for advanced gastric/GEJ adenocarcinoma, in which a single superficial endoscopic biopsy may underrepresent intratumoral heterogeneity or progressing metastatic niches. However, liquid biopsy does not preserve tissue architecture and cannot directly measure CD8+ T-cell entry into tumor nests, CAF/ECM barrier thickness, TLS maturity, LAMP3+ dendritic-cell proximity, or PD-1/PD-L1 spatial co-localization. A practical translational strategy may therefore combine baseline or progression spatial tissue profiling to define the dominant resistance ecology with longitudinal liquid biopsy to monitor temporal tumor evolution during therapy (118, 119).

## Clinical implementation and trial design: from spatial bottlenecks to trial hypotheses

Spatial immune ecology can inform rational combination strategies by identifying the dominant biological bottleneck, but spatial findings should be separated into established clinical contexts, trial rationales, and exploratory strategies (60). For immune-desert tumors, trials may test priming approaches such as immunogenic chemotherapy, radiotherapy, oncolytic viruses, vaccines, or innate immune agonists to initiate immune recruitment (17, 60). For immune-excluded tumors, vascular normalization, TGF-β blockade, extracellular-matrix remodeling, or myeloid reprogramming can be tested as ways to restore physical access (32, 33, 57). For inflamed-dysfunctional tumors, checkpoint combinations, regulatory T-cell modulation, suppressive myeloid targeting, dendritic-cell activation, or metabolic interventions may be most rational when the relevant suppressive niche is present (16, 38, 65). For TLS-rich immune-active tumors, strategies should preserve or enhance productive immune organization rather than indiscriminately deplete lymphoid or stromal elements (35, 36, 66). These examples are trial-development logic, not treatment recommendations for spatially selected gastric/GEJ cancer.

### Spatial ecology-guided combination therapy strategies

Spatial ecology can guide combination therapy design by specifying which tissue-level bottleneck the added agent is intended to remodel. In immune-excluded tumors dominated by a CAF/myeloid-cell barrier, candidate strategies include TGF-β pathway inhibition, extracellular-matrix remodeling, and selected myeloid-targeting approaches. The spatial pharmacodynamic goal is not generalized stromal depletion, but reduction or reprogramming of suppressive CAF/ECM and macrophage-rich neighborhoods so that effector T cells and antigen-presenting cells can enter malignant epithelial nests and interact productively (32, 33, 38, 57, 62, 86).

For tumors characterized by hypoxia and aberrant vasculature, anti-angiogenic or vascular-normalizing strategies provide a complementary remodeling logic. VEGF/VEGFR blockade may reduce endothelial anergy, improve perfusion and oxygenation, limit suppressive myeloid and regulatory-cell recruitment, and increase lymphocyte trafficking into tumor regions, thereby creating a spatial rationale for combining anti-angiogenic therapy with immune checkpoint blockade (16, 75, 87). In this setting, spatial readouts should test whether the combination actually decreases hypoxic macrophage-rich zones, improves tumor-nest T-cell penetration, and restores dendritic-cell/T-cell proximity rather than relying only on radiographic response.

Antigen-directed strategies can also be interpreted spatially. HER2- or CLDN18.2-directed antibodies, CLDN18.2-targeting antibody-drug conjugates, and related antigen-directed modalities may preferentially debulk antigen-positive tumor regions, promote local antigen release, and alter Fc-effector or payload-induced immune neighborhoods (42–44, 100, 120). However, heterogeneous HER2 or CLDN18.2 expression may generate antigen-negative or poorly accessible escape regions. Spatial profiling may therefore help define whether antigen-positive nests are physically accessible to effector mechanisms, whether adjacent immune neighborhoods are activated or suppressed, and whether post-treatment antigen loss, stromal exclusion, or myeloid dominance emerges as a resistance pattern. These applications remain trial-development hypotheses and pharmacodynamic endpoints until prospectively validated in gastric/GEJ ICI outcome-linked cohorts.

### Why mechanism-matched combinations may fail

A mechanism-matched combination may still fail. The dominant resistance niche can be under-sampled or can differ between primary and metastatic lesions. A pathway that appears dominant at baseline may be bypassed during therapy through redundant stromal, vascular, or myeloid circuits. Timing and sequence may also matter: priming, barrier remodeling, and checkpoint reinvigoration may need to occur in a coordinated order rather than as simultaneous additions. Toxicity can limit target inhibition, especially when checkpoint, antiangiogenic, stromal, and myeloid-targeting agents are combined. Finally, a spatially plausible target may be present but not in the compartment where it controls T-cell access or antigen presentation. These considerations argue for early pharmacodynamic tissue endpoints rather than reliance on response rates alone.

HER2-positive gastric cancer illustrates spatially integrated therapy, although current HER2-directed decisions remain based on established clinical biomarkers rather than spatial selection. From an ecological perspective, targeted agents can modify the microenvironment (100). Trastuzumab can mediate antibody-dependent cellular cytotoxicity (ADCC) and alter tumor-immune interactions by engaging Fc receptor-bearing effector cells such as macrophages and natural killer cells; chemotherapy may increase antigen release; and concurrent PD-1 blockade can sustain cytolytic function in responsive settings (9, 41, 100). Regional responses to HER2-PD-1 combinations may nevertheless be shaped by HER2 heterogeneity, macrophage and NK-cell positioning, and T-cell infiltration depth (46). Similar ecological logic may apply to CLDN18.2-targeted therapy, antiangiogenic agents, and antibody-drug conjugates (44, 100). Spatial profiling should generate pharmacodynamic and resistance hypotheses, not replace guideline-supported biomarker testing.

Peritoneal metastasis, a major and lethal dissemination route in gastric cancer, presents a highly organ-specific spatial ecosystem (22, 68). Its bottlenecks include baseline intraperitoneal immune suppression, lipid-rich suppressive ascites-associated myeloid cells, abnormal fibroblast-mesothelial crosstalk, poor physical T-cell access, and altered drug delivery dynamics, all of which may compromise systemic checkpoint blockade (59, 68). Single-cell studies of gastric cancer peritoneal metastases and ascites show enrichment of metabolically reprogrammed macrophages and pro-invasive stromal subsets that form a complex resistance network (101). Spatial profiling of peritoneal lesions, paired with single-cell analysis of ascites-derived states, may help identify niche-adapted combinations; possible trial concepts include localized myeloid metabolic modulation, targeting mesothelial-stromal interactions, intraperitoneal delivery to overcome systemic barriers, or engineered cellular therapies optimized for this environment (68, 101).

### Box 3. Temporal sampling logic for spatial resistance

- Primary resistance: baseline sampling should capture the invasive front or a metastatic lesion when feasible. Relevant spatial scenes include true immune deserts, pre-existing CAF/ECM barriers, antigen-presentation defects, and suppressive peritoneal or liver niches.
- Adaptive resistance: early on-treatment sampling can reveal induced PD-L1 at tumor-immune interfaces, expansion of Tregs or macrophages near effector cells, increasing hypoxia, or failure of progenitor-like T cells to expand.
- Acquired resistance: progression biopsies after prior response can identify immune-edited antigen-loss subclones, JAK/IFN pathway disruption, newly dominant stromal or vascular barriers, TLS disruption, or organ-specific escape in progressing lesions.
- Mixed response: paired sampling of responding and progressing lesions can distinguish lesion-intrinsic resistance from niche-driven escape, drug-delivery barriers, or spatial heterogeneity of HER2, CLDN18.2, PD-L1, or immune architecture.

A key translational principle is that spatial biomarkers should function not only as baseline predictors but also as pharmacodynamic endpoints in clinical trials (97, 102). Early-phase trials should ask whether a regimen remodels the intended niche: do effector T cells penetrate stromal barriers, are LAMP3+ mature dendritic cells recruited into relevant neighborhoods, is CAF-rich barrier thickness reduced, do TLSs mature or disorganize, and are hypoxic or macrophage-rich zones attenuated? (102). Two explicit gates should precede clinical implementation: independent external validation of a simplified marker panel and prospective testing of clinical utility in biomarker-linked trials. Baseline, on-treatment, and progression biopsies can then map the trajectory from initial phenotype to pharmacodynamic remodeling and acquired resistance (97, 102).

### Box 4. Practical implications for gastric/GEJ immunotherapy trials

The practical message for gastric/GEJ immunotherapy trials is that resistance should be understood, at least in part, as a tissue-ecological property. Single-cell atlases define the malignant, immune, stromal, vascular, and myeloid states involved, whereas spatial multi-omics explains how these states are assembled into productive, excluded, dysfunctional, lymphoid-organized, or metastatic ecosystems. This perspective helps reconcile why inflamed tumors can resist therapy when lymphocytes are trapped in stroma, why PD-L1 can mislead when expressed by spatially sequestered macrophages, why B-cell-rich tumors are not necessarily TLS-competent, and why responses can diverge across lesions within one patient (22–25, 31, 46, 75, 89).

- Baseline biopsies should preferentially include invasive-front or metastatic tissue when safe, because superficial endoscopic samples may miss CAF-rich barriers, TLSs, and organ-specific niches.
- TLS, CXCL13+ T-cell, LAMP3+ dendritic-cell, and CAF-myeloid features should be reported as investigational spatial markers unless validated in gastric ICI outcome-linked cohorts.
- Early on-treatment biopsies can serve as pharmacodynamic readouts by testing whether a regimen increases T-cell entry, restores APC-T-cell proximity, remodels CAF/ECM barriers, or reduces suppressive myeloid neighborhoods.

Accordingly, spatial feature frameworks, SICS-like composite concepts, and mechanism-matched combinations should be treated as conceptual prototypes or trial-development hypotheses until they are prospectively validated in gastric/GEJ ICI outcome-linked cohorts.

## Methodological pitfalls

Single-cell and spatial multi-omics are powerful but not self-interpreting. scRNA-seq can lose fragile cells, granulocytes, plasma cells, fibroblast subsets, and large epithelial cells during dissociation, and enzymatic digestion can induce stress-response artifacts. Spatial studies face additional complications from FFPE RNA degradation, epitope variability, necrosis, ulceration, mucin, tumor purity, and batch effects. These issues are particularly relevant in gastric cancer, where mucosal inflammation, diffuse-type infiltration, and treatment exposure can strongly affect tissue quality (103, 104).

Spatial platforms answer different questions. Spot-based transcriptomics supports broad discovery but mixes multiple cells per spot. High-resolution *in situ* transcriptomics provides cellular or subcellular localization but usually relies on selected gene panels. Spatial proteomics offers clinically interpretable protein localization, with dependence on antibody quality, epitope preservation, and segmentation. GeoMx-like region profiling is FFPE-compatible and scalable, although region-of-interest selection can introduce bias. Technology selection should therefore follow the biological question, and large technology-comparison tables are provided as **Supplementary Table 4** rather than in the main PDF (56, 103–105).

Beyond computational inference, spatial technologies also have hardware- and resolution-dependent limitations. Spot-based transcriptomic platforms offer broad molecular coverage and tissue-scale discovery, but their effective resolution may be limited by spot diameter, transcript diffusion, capture efficiency, and the presence of multiple cells within one capture area. *In situ* transcriptomic and multiplex imaging platforms improve cellular or subcellular localization, but they are constrained by field of view, imaging throughput, optical resolution, autofluorescence, signal crowding, panel design, antibody or probe validation, and segmentation quality. Region-of-interest platforms reduce tissue and cost burden but depend on pathologist-guided ROI selection and may miss unanticipated microdomains. Across platforms, tissue thickness, fixation, decalcification when relevant, FFPE RNA degradation, epitope preservation, scanner calibration, batch effects, data-storage demands, and cross-platform harmonization can all affect reproducibility. These constraints should be reported alongside computational pipelines so that spatial claims are interpreted as platform-dependent measurements rather than technology-neutral biological facts (56, 103–105).

Computational inference also needs restraint. Ligand-receptor tools predict possible communication from transcript co-expression; they do not prove protein binding or active downstream signaling. Deconvolution depends on the quality of the reference atlas, and neighborhood analyses depend on segmentation accuracy, distance thresholds, two-dimensional section orientation, and region annotation. Spatial hypotheses should be checked by orthogonal methods such as multiplex immunofluorescence, independent cohorts, and functional perturbation models whenever feasible (106–108).

Several interpretive pitfalls should be stated explicitly. TLSs are not uniformly favorable; PD-1 expression may mark activation, tumor reactivity, exhaustion, or bystander stimulation; CAFs can restrain tumor spread as well as exclude lymphocytes; prognostic value is not the same as predictive value; and superficial biopsies may miss the invasive front or metastatic ecology. Before spatial biomarkers are used for routine selection, studies should report tissue region, platform, resolution, cell segmentation, cell annotation, treatment history, and clinical endpoint (97, 99, 109, 110).

## Discussion and outlook

The central message of this Review is that immunotherapy resistance in gastric/GEJ adenocarcinoma should be understood, at least in part, as an ecological property of tissue. Single-cell atlases define the participating malignant, immune, stromal, vascular, and myeloid states, whereas spatial multi-omics explains how those states are arranged into productive, excluded, dysfunctional, lymphoid-organized, or metastatic ecosystems. This interpretation helps reconcile why highly inflamed tumors can resist therapy when lymphocytes are trapped in stroma, why PD-L1 can mislead when expressed by spatially sequestered macrophages, why B-cell-rich tumors are not necessarily TLS-competent, and why responses can diverge across lesions within one patient (22–25, 31, 46, 75, 89).

Spatial immune ecology contextualizes current biomarkers rather than replacing them. PD-L1 CPS or TAP score, MSI/dMMR, HER2, and CLDN18.2 remain central to clinical stratification in appropriate treatment settings, whereas EBV status and TMB provide important biological or supportive context. Their interpretation depends on the tissue architecture in which they are embedded. A high PD-L1 CPS driven by a macrophage-rich, CAF-dense stromal barrier implies a different resistance biology from PD-L1 expression at an active interface between malignant cells and clonally expanded CD8+ T cells. Similarly, MSI-high status signals antigenic potential, but spatial exclusion or local antigen-presentation failure may still restrict response (12, 38, 40, 57, 75).

The translational opportunity is to match the dominant spatial bottleneck to a testable intervention. Immune deserts can be studied with priming strategies; immune-excluded tumors with barrier, vascular, or myeloid remodeling; inflamed-dysfunctional tumors with suppression-relieving combinations; TLS-rich tumors with immune-preserving or immune-amplifying approaches; and peritoneal or liver metastases with niche-specific strategies. These are not standards of care for spatially selected gastric/GEJ cancer, but hypotheses to be tested with baseline, on-treatment, and progression biopsies as pharmacodynamic endpoints (59, 68, 97, 102, 109–112).

The field now needs to move from descriptive atlases to longitudinal, outcome-linked, globally representative validation. Future cohorts should include intestinal and diffuse histologies, EBV-positive and MSI-high tumors, HER2- and CLDN18.2-positive disease, heavily pretreated and treatment-naive samples, Asian and non-Asian populations, primary and metastatic sites, and adequately annotated GEJ adenocarcinomas. Public reference atlases, standardized region annotation, open analysis pipelines, AI-assisted pathology grounded in molecular truth, and transparent reporting guidelines will be essential if spatial immune ecology is to mature from a conceptual framework into clinically reliable precision oncology (80, 99, 109, 113–117).

Until that evidence base is in place, spatial feature frameworks, SICS-like composite concepts, and mechanism-matched combinations should remain conceptual prototypes or hypothesis-generating strategies, even when the underlying biology is compelling.

## Conclusion

Spatial immune ecology interprets immunotherapy resistance in gastric/GEJ adenocarcinoma through tissue-level immune access, immune competence, and immune suppression. By integrating single-cell states with spatial coordinates, this framework explains how similar molecular biomarkers can coexist with different immune architectures and clinical trajectories. Its most immediate translational value is not to replace PD-L1 CPS/TAP, MSI/MMR, HER2, CLDN18.2, or other guideline-supported tests, but to complement them as lesion-level explanatory biomarkers and pharmacodynamic endpoints. Progress will depend on standardized sampling, lean validated panels, independent external validation, GEJ-annotated cohorts, and prospective trials that test whether spatially defined bottlenecks can be remodeled into durable antitumor immunity.

## Supplementary material

Supplementary Table 1 summarizes key concepts and working definitions in gastric/GEJ spatial immune ecology.

Supplementary Table 2 summarizes representative single-cell, immune-profiling, and spatial multi-omics studies.

Supplementary Table 3 summarizes spatial immune-ecological phenotypes and hypothesis-supported therapeutic implications.

Supplementary Table 4 compares technologies for studying gastric cancer spatial immune ecology.
