# Supplementary material for: Spatial immune ecology of immunotherapy resistance in gastric and gastroesophageal junction adenocarcinoma
Source: Front Oncol. 2026 Jul 21;16:1895422. doi: 10.3389/fonc.2026.1895422 (PMC13433203; doi:10.3389/fonc.2026.1895422)
Supplement: Supplementary file 1 [file Table1.docx]

# Supplementary Tables for Spatial immune ecology of immunotherapy resistance in gastric and gastroesophageal junction adenocarcinoma

## Supplementary Table S1. Key concepts and working definitions in gastric/GEJ spatial immune ecology

| **Concept** | **Working definition** | **Spatial readout** | **Relevance to immunotherapy resistance** | **Representative markers** | **Validation methods** |
| --- | --- | --- | --- | --- | --- |
| Immune accessibility | Physical ability of lymphocytes and antigen-presenting cells to enter malignant compartments | Intratumoral vs stromal CD8/DC density; distance to tumor nests; vascular access | Low accessibility can cause immune exclusion despite inflammatory signals | CD8A/CD8, ITGAX/DC markers, vascular markers, tumor-nest segmentation | mIF/IMC, spatial transcriptomics, tumor-stroma segmentation, nearest-neighbor analysis |
| Immune competence | Capacity of local immune networks to present antigen and sustain tumor-reactive T cells | TCF7+ or CXCL13+ T cells; LAMP3+ DC proximity; TLS maturity; TCR clonality | Competent niches may indicate reinvigoratable immunity after PD-1 blockade | TCF7, CXCL13, LAMP3, CD80, TCR clonality, TLS markers | scRNA-seq plus spatial validation, scTCR-seq, TLS maturity scoring, multiplex imaging |
| Immune suppression | Local dominance of inhibitory cells, ligands, stromal barriers, and metabolic stress | Treg/CD8 ratio; macrophage-rich neighborhoods; CAF/ECM barriers; hypoxic zones | Suppressive niches can overcome antigenicity and PD-L1 positivity | FOXP3, CTLA4, SPP1, TGFB1, CXCL12, COL1A1, hypoxia markers | Spatial proteomics, CAF/myeloid neighborhood analysis, hypoxia staining, ligand-receptor validation |
| Immune desert | Low immune infiltration in tumor and stroma | Low CD8, low DC, low IFN-gamma, absent TLS | May require priming or antigenicity-enhancing strategies | Low CD8/GZMB/IFNG, low DC markers, absent TLS features | Multi-region sampling, CD8/DC IHC or mIF, spatial transcriptomic confirmation |
| Immune exclusion | T cells present but physically retained outside malignant nests | High stromal CD8 with low tumor-nest CD8; CAF/ECM boundary | May require access-restoring stromal, vascular, or myeloid modulation | Stromal CD8, COL1A1/FAP/ACTA2, CXCL12/TGFB1 boundary | Invasive-front sampling, collagen/CAF staining, spatial CD8 compartment analysis |
| TLS-rich immune-active niche | Organized B-T-DC neighborhoods with HEV-like features and local antigen presentation potential | Mature TLS; CXCL13+ T cells; GC B cells; LAMP3+ DC; HEV markers | May complement PD-L1/MSI as a candidate response-associated architecture | CD20/CD79A, BCL6, CXCL13, LAMP3, HEV markers | TLS maturity scoring, germinal-center markers, HEV markers, outcome-linked validation |

## Supplementary Table S2. Representative single-cell, immune-profiling, and spatial multi-omics studies informing gastric/GEJ immune ecology

| **Study** | **Approach** | **Samples/data** | **Key insight** | **Resistance relevance / limitation** | **DOI** | **Evidence tier / ICI outcome-linked?** |
| --- | --- | --- | --- | --- | --- | --- |
| Zhang et al., Cell Reports 2019 | scRNA-seq of premalignant lesions and early gastric cancer | 32,332 cells from gastric lesions | Lineage remodeling and immune/stromal changes during early tumorigenesis | Suggests ecology begins before advanced cancer; not ICI-treated cohort | 10.1016/j.celrep.2019.04.052 | No; discovery/biology cohort |
| Sathe et al., Clin Cancer Res 2020 | Single-cell genomic characterization of gastric TME | Primary gastric cancer samples | Reprogrammed stromal, macrophage, dendritic, Treg, and exhausted T-cell states | Identifies candidate checkpoint and communication axes; limited spatial context | 10.1158/1078-0432.CCR-19-3231 | No; discovery/biology cohort |
| Wang et al., Nat Med 2021 | scRNA-seq of metastatic gastric adenocarcinoma | Peritoneal carcinomatosis samples | Lineage diversity and metastatic microenvironmental remodeling | Highlights organ-specific resistance; spatial validation needed | 10.1038/s41591-020-1125-8 | No; discovery/biology cohort |
| Kumar et al., Cancer Discovery 2022 | Large single-cell atlas | >200,000 cells from 48 samples | Lineage states, TME programs, and subtype-specific expression patterns | Links molecular subtype to ecosystem state; not primarily spatial | 10.1158/2159-8290.CD-21-0683 | No; discovery/biology cohort |
| Ma et al., Cancer Discovery 2025 | Spatial transcriptomics + scRNA-seq | Spatial ROIs and single-cell profiles across gastric adenocarcinoma samples | Spatial ITH associated with immune microenvironments and checkpoint profiles | Directly connects spatial tumor states to resistance biology; needs outcome validation | 10.1158/2159-8290.CD-24-0605 | No; spatial/prognostic or biology evidence |
| Wang et al., Nat Commun 2025 | Single-cell and spatial transcriptomics focused on TLS | 30 gastric cancer specimens stratified by TLS patterns | Immune-active TLS enriched for CXCL13+ T cells, GC B cells, LAMP3+CD80+ DCs, HEV-associated cells | Provides TLS-based spatial signature; predictive use requires prospective cohorts | 10.1038/s41467-025-65421-8 | No; spatial/prognostic or biology evidence |
| Gao et al., Nat Commun 2026 | Spatially resolved atlas of gastric cancer | Single-cell and spatial transcriptomic data from gastric cancer donors | Defines lymphocyte-aggregated region and PD-1+CD27+CD8 T cell proximity to CD70+LAMP3+ DCs | Suggests productive immune neighborhoods; functional proof remains needed | 10.1038/s41467-026-68612-z | No; spatial/prognostic or biology evidence |
| Mao et al., Front Immunol 2026 | Integrated single-cell, spatial, and bulk transcriptomics | Public and validation datasets | CAF-centered collagen-integrin/MIF signaling and FMR1-FTO axis linked to immune exclusion | Nominates stromal resistance axis; requires therapeutic validation | 10.3389/fimmu.2026.1713267 | No; spatial/prognostic or biology evidence |
| Cousin et al., Mol Cancer 2024 | Spatial transcriptomics + plasma proteomics | Gastric cancer cohort treated with anti-PD-1/PD-L1 + antiangiogenesis | Spatial and plasma proteomic features associated with primary resistance | Outcome-linked evidence; combination-therapy context and prospective validation needed | 10.1186/s12943-024-02092-x | Yes; gastric ICI outcome-linked, combination-therapy context |

## Supplementary Table S3. Spatial immune ecological phenotypes and hypothesis-supported therapeutic implications

| **Phenotype** | **Spatial features** | **Candidate biomarkers** | **Dominant bottleneck** | **Trial-development hypothesis (not a treatment recommendation)** |
| --- | --- | --- | --- | --- |
| Immune desert | Low immune infiltration in tumor and stroma; weak DC/TLS presence | Low intratumoral CD8, low IFN-gamma, low DC recruitment, absent TLS | Priming and recruitment failure | Trial hypothesis: chemotherapy/radiotherapy priming, vaccines, innate agonists, oncolytic therapy, or adoptive therapy after biomarker validation |
| Immune excluded | T cells in stroma or margin but absent from tumor nests; CAF/ECM/myeloid barriers | High stromal CD8 with low tumor-nest CD8; TGF-beta/CAF signature; collagen boundary | Physical access failure | Trial hypothesis: PD-1-based therapy plus vascular, stromal, matrix, or myeloid modulation after spatial validation |
| Inflamed dysfunctional | Intratumoral lymphocytes with exhaustion, Tregs, macrophages, inhibitory ligands, metabolic stress | PD-1+TIM-3+/LAG-3+/TIGIT+ T cells; Treg proximity; PD-L1 co-localization; IDO/hypoxia | Functional reinvigoration failure | Trial hypothesis: checkpoint combinations, Treg/myeloid/metabolic modulation, or DC activation after spatial validation |
| TLS-rich immune-active | Organized B-T-DC niches; CXCL13+ T cells; GC B cells; HEV-like cells | Mature TLS score, LAMP3+ DC proximity, CXCL13, CXCR5, CD70-CD27 axis | Immune competence preservation/enhancement | Trial hypothesis: PD-1-based trial context after validation; TLS-preserving or TLS-enhancing strategies; DC activation |
| Peritoneal/metastatic niche | Organ-specific stromal, mesothelial, macrophage, and ascites-associated suppression | Mesothelial-fibroblast interaction, macrophage-rich ascites, discordant primary-metastatic immune biomarkers | Organ-specific immune access and suppression | Trial hypothesis: local delivery, myeloid/stromal targeting, cellular therapy, or niche-specific combinations after metastatic-site validation |

## Supplementary Table S4. Technologies for studying gastric cancer spatial immune ecology

| **Modality** | **Best use** | **Strengths** | **Limitations** | **Translational readiness** | **FFPE compatibility** | **Clinical lab suitability** |
| --- | --- | --- | --- | --- | --- | --- |
| scRNA-seq/snRNA-seq | Cell-state discovery across malignant and non-malignant compartments | Unbiased transcriptome-wide cell-state identification | Loses spatial context; dissociation/nuclei bias | Discovery; requires spatial validation | Fresh/frozen preferred; snRNA-seq can use frozen tissue | Discovery; not routine clinical testing |
| scTCR-seq/scBCR-seq | Immune clonality and lineage relationships | Links state to clonal expansion and possible antigen-driven response | Does not prove antigen specificity; needs localization | Discovery/biomarker refinement | Fresh/frozen preferred | Discovery/refinement |
| CITE-seq/CyTOF | Protein-informed immune phenotyping | Improves checkpoint and activation marker detection | Panel constraints; tissue dissociation | Discovery and validation | Fresh tissue preferred; CyTOF can use selected fixed workflows | Translational immune profiling |
| Spot-based spatial transcriptomics | Whole-section regional mapping | Broad gene coverage with histology | Multi-cell spots; deconvolution needed | Discovery; limited routine use | FFPE-compatible platforms available | Discovery and assay development |
| High-resolution in situ transcriptomics | Single-cell/subcellular mapping of transcripts | Direct neighborhood and proximity analysis | Panel/chemistry constraints; cost | Discovery and targeted validation | FFPE-compatible options available | Targeted validation after panel reduction |
| GeoMx/region-based profiling | Tumor-stroma-TLS-invasive front comparisons | FFPE-compatible; region-directed | Region selection bias; not true single-cell | Translational validation | Strong FFPE compatibility | Translational validation in selected labs |
| Multiplex IF/IMC/CODEX/MIBI | Protein-level spatial validation | Clinically interpretable markers; strong for architecture | Antibody validation and image-analysis variability | High translational potential with standardized panels | FFPE-compatible with antibody validation | High potential with standardized panels |
| Digital pathology and AI | Scalable extraction of morphological spatial features | Potentially deployable on routine slides | Requires external validation and interpretability | Potential clinical deployment after validation | Routine H&E/IHC FFPE slides | Potential deployment only after external validation and assay-standardization |
